# Structural–Phase Transformations in Stainless Steel CF8 Under Ion Implantation and Thermal Treatment

**DOI:** 10.3390/ma18215062

**Published:** 2025-11-06

**Authors:** Irina Manakova, Mikhail Vereshchak, Gaukhar Yeshmanova, Zhandos Tleubergenov

**Affiliations:** Institute of Nuclear Physics, Ibragimov St. 1, Almaty 050032, Kazakhstan

**Keywords:** stainless steel, ion implantation, Mössbauer spectroscopy

## Abstract

The γ ⟶ α′-transformation under implantation of austenitic-ferritic steel CF8 with ^57^Fe ions to fluences of 1 × 10^16^, 5 × 10^16^ and 1 × 10^17^ ion/cm^2^, as well as the reverse α′ ⟶ γ–transformation under thermal treatment, was studied using transmission Mössbauer spectroscopy (MS), conversion electron Mössbauer spectroscopy (CEMS), and X-ray diffraction (XRD) methods. It was found that implantation, which causes radiation damage with 36 dpa, results in the formation of α′-martensite. At higher fluences of implanted ions, the amount of α′-martensite increased, reaching 86 at.% within the irradiated layer. Annealing in the temperature range of 600–850 °C resulted in the observed reverse transformation of α′-martensite to γ-austenite. The dependence of the average effective magnetic field on the annealing temperature was established.

## 1. Introduction

The development of structural materials for designed nuclear facilities is a nontrivial scientific and engineering challenge [1,2,3]. At present, a wide set of performance requirements is imposed on such materials [3,4,5]. Structural materials intended for use under complex radiation and temperature exposure require increased radiation resistance, phase stability, corrosion resistance, and high impact toughness for a long service life [2,6,7]. Traditionally, austenitic and ferritic-martensitic steels have been used in the nuclear industry [7,8]. Modern approaches to developing materials for nuclear power are focused on actively managing phase metastability through controlled phase transformations [9,10,11,12,13]. In parallel, the research field of oxide dispersion-strengthened ferritic-martensitic steels and nanostructured alloys is advancing [14,15,16]. The development of high-entropy alloys with a multiphase structure, capable of combining radiation and corrosion resistance with high ductility, is also underway [17,18]. Nevertheless, austenitic-ferritic (duplex) stainless steels, which have an optimal combination of strength, ductility, and corrosion resistance, remain among the materials used in the nuclear industry. One representative of the duplex steel class is CF8 steel, widely used in components of nuclear power plants operating in an atmosphere of extreme pressure, such as primary coolant piping, valve bodies, and some internal reactor core components. However, with prolonged exposure to radiation and temperature, complex processes of radiation-induced phase transformations occur in structural materials, accompanied by a redistribution of components and a change in short-range order [2,19,20]. High radiation doses produce damage in the near-surface layer that determines the local surface structure [21,22]. Much attention is currently being paid to spinodal decomposition and radiation-induced ordering processes in the Fe–Cr systems [23,24,25]. These phenomena determine changes in chemical heterogeneity and magnetic properties, and also affect the corrosion resistance and brittleness of these materials [23,26,27]. Earlier studies also reported irradiation-induced spinodal decomposition in different types of compounds [28,29]. In particular, enhanced spinodal decomposition was observed in Fe–32%Cr alloy irradiated with neutrons to 0.03 dpa at 290 °C [28]. According to [30], the formation of areas with local enrichment of chromium can occur under both thermal and radiation influences, which accelerate diffusion processes and stabilize nonequilibrium states of short-range order.

Despite significant advances in understanding the factors driving these phase transformations, the mechanisms underlying the formation of short-range order (SRO) during ion implantation, as well as the mechanisms of its evolution following thermal treatment, remain poorly understood. Studies [31,32,33,34] have demonstrated that even low doses of ion irradiation can trigger a redistribution of atoms in the tens of nanometer range, leading to the formation of regions with altered short-range order. The nature of these changes depends significantly on the chemical composition of the alloy, the implantation temperature, and the energy parameters of the ions. This stage therefore requires careful control of such process criteria as ion energy, concentration, and implantation time.

Ion implantation is an effective tool for modeling radiation effects, providing a controlled method for introducing defects and alloying atoms into the near-surface layer. The introduction of ^57^Fe ions allows the use of Mössbauer spectroscopy methods for subsequent detailed analysis of structural evolution [35,36]. This method can provide information on the electronic structure of implanted atoms, their location in the host matrix and the environment, the annealing of crystal lattice defects, aggregation processes, etc. Previously, the authors attempted to study the effect of ^57^Fe ion implantation on structural changes in metallic Ta and Mo, as well as in 12Cr18Ni10T stainless steel, using Mössbauer spectroscopy [37,38]. Additionally, radiation damage in Nb-Zr alloys was also studied using Mössbauer methods [39].

Of additional interest is the effect of ion implantation on the mechanical and corrosion properties of duplex steels. [40,41]. According to [23], changes in short-range order and the formation of finely dispersed regions of the α′-phase can lead to material strengthening and a change in magnetic susceptibility. Depending on the nature of the subsequent heat treatment, either the dissolution of defects and restoration of the original state or further phase decomposition can occur. Studies [42,43] have shown that the thermal stability of irradiated alloys is determined by the balance between the rate of diffusion decomposition and defect recombination, which is of particular importance when operating materials at unstable temperatures.

When a uniform surface layer is formed, the service life of implanted samples can be extended [44]. It is known that austenitic alloys are susceptible to martensitic transformations and the associated surface strengthening under thermal and radiation exposure. However, in austenitic steels, martensitic structures generally show poorer resistance to aggressive environments, the formation of which depends on the ambient temperature and irradiation conditions [45]. At the same time, irradiation can simultaneously cause multiple processes, in particular, diffusion and segregation of dissolved components, radiation-induced disordering or amorphization [46,47,48].

Previous studies showed that implantation with metalloids can lead to formation of either stable or metastable metal-metalloid phases [49], while the implantation of carbon ions leads to the formation of carbide compounds [50]. In [50], it was found that implantation of carbon ions into austenitic stainless steel AISI 316L, besides the austenite-to-ferrite phase transformation, can form an amorphous layer and nanophase precipitation at different implantation doses.

Carbide formation plays a key role for the performance characteristics of steel (hardness, impact toughness, wear resistance, corrosion resistance, etc.). Carbide hardness is higher than that of stainless steel. Consequently, this provides increased wear resistance. However, excessive carbide formation under certain conditions can degrade the impact toughness and corrosion resistance of steel. The effect of microsegregation on the formation of M_7_C_3_ carbides was studied in [51,52,53]. According to the model proposed in [51], microsegregation of alloying elements promotes an increase in the Cr content in the alloy structure due to differences in the thermodynamic equilibrium of element solubility in different phases. This leads to the formation of heterogeneous nuclei and the subsequent growth of bulk carbides. As the proportion of the solid phase increases, more Cr is consumed for carbide formation, leading to a decrease in its content in the alloy. At the same time, microsegregation promotes the evolution of the morphology of eutectic carbides.

As demonstrated in [54,55] chromium segregation is a key factor leading to the degradation of grain boundary corrosion resistance. Chromium segregation alters the local phase composition, which directly impacts the alloy’s phase stability. A decrease in chromium concentration can lead to local instability of the passive oxide film that protects the steel from corrosion. This leads to the initiation of intergranular and pitting corrosion [56,57], especially under the influence of radiation-induced processes.

The accumulation of defects due to cascade atom sputtering leads to the destruction of the original short-range order due to radiation mixing and the formation of a new short-range order corresponding to the modified local composition [58,59]. Study [23], describing the stability of modified alloys, showed that upon subsequent heating, defect-enriched zones become centers of accelerated spinodal decomposition. Consequently, implantation changes not only the initial state but also the surface energy, influencing the direction of subsequent phase transformations.

Thermal treatment is the main factor influencing the behavior of defect configurations and short-range order. Studies [58,60] showed that low-temperature treatment leads to partial restoration of short-range order and defect structure, while high-temperature treatment induces the diffusion processes, which can result in the formation of Cr-enriched regions and carbide phases. The combination of ion implantation and subsequent controlled thermal treatment enables the evaluation of short-range order evolution as a stage of structural rearrangement that determines the macroscopic properties of the alloy.

Despite the extensive data on phase transformations in steels, comprehensive studies combining ion implantation, annealing, and local surface analysis methods are insufficient for CF8 steels using Mössbauer spectroscopy. The need to establish patterns of implantation influence on short-range order and phase stability stems from both the fundamental interest in radiation-induced structural and phase transformations and the practical value of this approach for assessing the service life of materials used in nuclear power engineering. The goal of this study is to investigate the influence of ^57^Fe ion implantation on phase transformations, short-range atomic order, and the subsequent evolution of the modified layer during annealing in CF8 steel by Mössbauer spectroscopy methods.

## 2. Materials and Methods

The Argonne National Laboratory (USA) provided samples of aged CF8 steel in the form of plates 10 mm × 10 mm in size and 600 μm thick for this study. Aging was performed according to the following scheme: heating to 350 °C and exposure at the specified temperature for 10,000 h. The steel samples were then ground, polished and rolled to a thickness of 20 μm. Thermal treatment at 850 °C for 4 h in a vacuum of 1 × 10^−6^ mm Hg was also used for Mössbauer and X-ray structural studies. Implantation of ^57^Fe ions was carried out on an electrostatic charge-exchange accelerator of heavy ions UKP-2-1 (Almaty, Kazakhstan). During implantation, the ion current density was 100 nA, the continuous ion beam energy was 200 keV, and the fluences were 1 × 10^16^, 5 × 10^16^ and 1 × 10^17^ ons/cm^2^. A schematic of the implantation setup is illustrated in Figure S1 (Supplementary Materials). The SRIM-2013 program [61] was used to calculate the degree of the ion beam impact on the steel crystal lattice. The average projective range of ^57^Fe ions was ~ 70 nm, the number of displacements per atom (dpa) was 36, 168 and 336 for fluences of 1 × 10^16^, 5 × 10^16^ and 1 × 10^17^ ion/cm^2^, respectively. This leads to a localized surface modification that has no macroscopic effect on the bulk structure but creates conditions for localized phase transformations in the modified layer. The implantation temperature is 60 °C, so the bulk structure and properties of the sample remain almost unchanged. The steel samples implanted with ^57^Fe ions at a fluence of 5 × 10^16^ ion/cm^2^ were subsequently subjected to isochronous annealing at temperature range of 300–850 °C. During annealing, phase transformations also occur in the near-surface layer. Subsequent annealing initiates the reverse α-γ transformation and can redistribute the implant. A thickness of 20 μm allows researchers to observe structural transformations of the studied surface layer within 100 nm over the entire annealing temperature range.

Transmission Mössbauer spectroscopy (MS) is one of the methods most widely used to study the chemical state of iron in the structure of iron-containing materials throughout the sample [62,63,64]. In addition to MS, the method of conversion electron Mössbauer spectroscopy (CEMS) was used to study the surface layers of the material and obtain information about a layer up to ~100 nm thick [65,66,67,68,69]. This method was very useful because the projected range of the implanted ions is commensurate with the escape depth of conversion electrons. MS and CEMS spectra were recorded at room temperature in constant acceleration mode using an MS-1104Em spectrometer (Research Institute of Southern Federal University, Rostov-on-Don, Russia). ^57^Co source in a chromium matrix served as the γ-ray source. Conversion electrons were detected with a gas proportional counter filled with a He + 8% CH_4_ mixture. Spectra were processed using the SpectrRelax software package (Version 2.4, Lomonosov Moscow State University, Moscow, Russia) [70]. Since the Mössbauer spectrum provides information on the nonequivalent positions of the ^57^Fe Mössbauer probe in the structure [32,71,72], the integral spectrum is a superposition of several subspectra from Fe atoms with different surroundings of alloying elements. Therefore, the distribution was further used to approximate the experimental spectra with a set of several magnetically split subspectra corresponding to different environments of ^57^Fe atoms by impurity atoms (primarily Cr and Ni). The parameters of the hyperfine interaction of the ^57^Fe nucleus characterizing the subspectra were: the isomer shift relative to metallic iron Is, the quadrupole splitting ∆, the quadrupole shift Qs for magnetically split spectra, the magnetic field H, full width at half maximum W and the integrated intensity of the subspectrum S. The Voigt pseudo-function was used as a hardware function describing the Mössbauer spectra.

The crystal structure of the studied samples was determined using X-ray diffraction analysis (XRD). The measurements were carried out on a D8 ADVANCE diffractometer (Bruker, Karlsruhe, Germany) with a Cu anode, operating parameters of 40 kV, 40 mA, increment 0.02°, and scanspeed 1.0 sec/stp. The phase analysis was carried out in the EVA program with an integrated ICDD database.

The elemental composition of the near-surface layer was determined by scanning electron microscopy (SEM) using a Hitachi TM4000Plus microscope (Hitachi High-Tech Corporation, Tokyo, Japan) equipped with an Quantax 75 energy-dispersive analyzer with an energy resolution on the characteristic radiation line Mn Kα_1,2_ of 137 eV at an accelerating voltage of 5–15 kV and an accumulation time of 60 s. The elemental composition of duplex stainless steel CF8 is given in Table 1.

## 3. Results

### 3.1. Ion Implantation of ^57^Fe Ions

**Figure 1:**
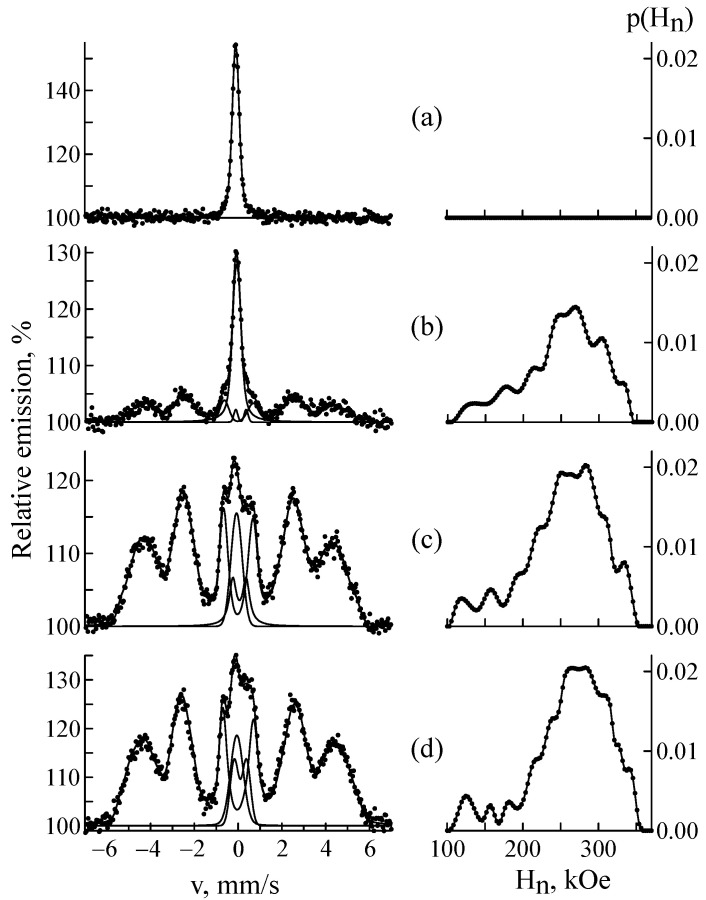
CEMS spectra and distributions of the hyperfine magnetic field p(H_n_) in the CF8 steel samples before (**a**) and after ^57^Fe ion implantation at a fluence of 1 × 10^16^ (**b**), 5 × 10^16^ (**c**) and 1 × 10^17^ ion/cm^2^ (**d**).

**Figure 2:**
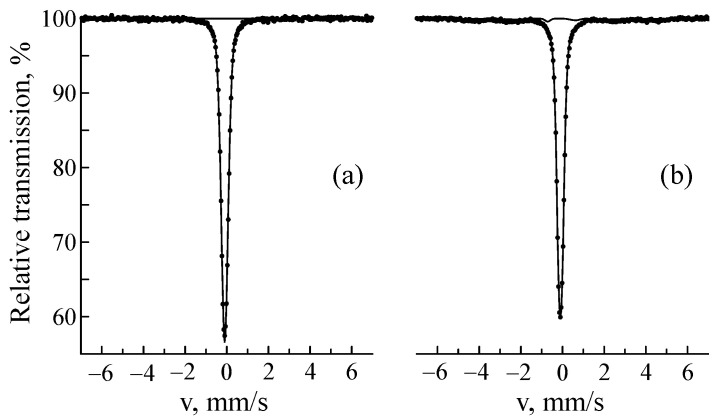
MS spectra of CF8 steel samples before (**a**) and after ^57^Fe ion implantation at a fluence of 1 × 10^17^ ion/cm^2^ (**b**).

Figure 1 shows the Mössbauer spectra in the electron backscattering geometry and the distribution of the hyperfine magnetic field p(H) of steel samples before and after implantation. Before implantation, the CEMS spectrum was a line of γ-austenite with an fcc lattice. The austenite spectrum can be processed by a doublet and/or a singlet. This is due to the fact that in austenite, substitution and introduced elements (mainly Cr and Ni) break the cubic symmetry at the iron position, which leads to the distribution of quadrupole splitting and isomer shift and thus spectrum broadening [45,68,73]. In our case, the CEMS spectrum of the sample before implantation was well approximated by a doublet with the parameters Is = −0.099 ± 0.005 mm/s, Δ = 0.13 ± 0.01 mm/s, and W = 0.325 ± 0.009 mm/s, which nearly coincided with the parameters of the transmission spectrum: Is = −0.099 ± 0.001 mm/s, Δ = 0.16 ± 0.01 mm/s, and W = 0.320 ± 0.003 mm/s (Figure 2a). The implantation of ^57^Fe ions led to the appearance of a sextet corresponding to α′-martensite. In the MS spectra of the sample implanted at a fluence of 1 × 10^16^ ion/cm^2^, this phase could not be isolated, but it amounted to 4.6 ± 0.3% at a fluence of 5 × 10^16^ ion/cm^2^ and 7.4 ± 0.3% at a fluence of 1 × 10^17^ ion/cm^2^ (Figure 2b). The weak influence of implantation on MS spectra is explained by the small increase in the average ^57^Fe content over the sample volume (from 0.01 at.% at 1 × 10^16^ ion/cm^2^ to 3.8 at.% at 1 × 10^17^ ion/cm^2^). The paramagnetic part of the CEMS spectra of the implanted samples could not be described by the model used for fitting the CEMS spectra of the samples before implantation. In addition to the doublet corresponding to γ-austenite, the CEMS spectra of the implanted samples contained a subspectrum/subspectra with a positive isomer shift. We cannot exclude the superposition of the austenite subspectrum with other paramagnetic (secondary) components arising during sample implantation. Therefore, a doublet with an isomer shift of Is = 0.10 ± 0.03 mm/s and a quadrupole splitting of Δ = 0.56 ± 0.02 mm/s was added to the model. According to [74], a doublet with Is = 0.08 mm/s and Δ = 0.47 ± 0.01 mm/s corresponds to chromium carbide Me_7_C_3_ (where Me = Fe, Cr) with a hexagonal crystal lattice [75]. In addition to this carbide, the most likely formation is Me_23_C_6_, the Mössbauer spectrum of which is a superposition of a doublet and a singlet with positive isomer shifts [32,76]. Thus, the doublet we added can reflect a combination of several carbides. From Figure 1 it is clear that the total amount of the supposed carbide phase grows with the increasing fluence.

**Figure 3:**
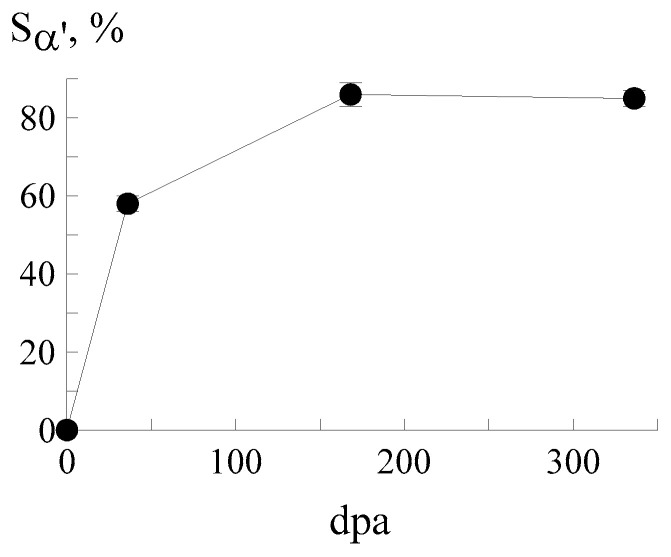
Relative intensity of α′-martensite in the CEMS spectra of CF8 steel as a function of dpa.

The most prominent implantation effect was the appearance of the magnetic component of the spectrum corresponding to the α′-phase with a bcc crystal lattice. Its content increased from ~58 to ~86% with the growing fluence. The dependence of the relative intensity of α′-martensite on dpa in the CEMS spectra of a CF8 steel sample is shown in Figure 3. Martensite formation is most intense at low dpa values (less than 36). At the interval of 36–168 dpa, the process slows down and then it stabilizes. Table 2 shows the quantitative phase composition of the samples after implantation with ^57^Fe ions at different fluences. As is known, the area under the curve of the integral profile of subspectrum S is proportional to the Mössbauer effect probability and to the number of iron atoms in nonequivalent positions [32,71,72]. It corresponds to the percentage content of phases with some ambiguity determined by the different Debye-Waller factors for different crystal lattices. This ambiguity can be neglected for comparison of the austenitic and martensitic components of the spectrum [75,77]. However, carbides have stronger interatomic bonds and, accordingly, the area under carbide doublets can appreciably exceed their percentage content [75]. Table 2 shows that the α′-martensite content remains relatively stable at high doses (of about 85–86%). This is because the depth of radiation damage is limited by the ion range. Therefore, at fluences greater than 5 × 10^16^ ion/cm^2^, the layer in which α′-martensite can form is already fully saturated. Therefore, the formation of new martensite regions is impossible. Additional ions accumulate in the already transformed region, only slightly affecting the proportion of α′-martensite. Furthermore, ion implantation at high doses can cause surface recrystallization, leading to the transformation of some α′-martensite into γ-austenite, which will lead to a decrease in the α′-martensite content.

The analysis of the fluence dependence of hyperfine parameters of the implanted samples showed that the average magnetic field <H> rises from ∼251 to ∼264 kOe with increasing fluence. Higher hyperfine fields correspond to lower chromium content, which is consistent with the results of [31], where the authors observed a decrease in the chromium content at high irradiation doses. This effect can be due to the segregation of chromium compounds (atoms) under the action of ^57^Fe ion flows. During ion implantation, the implant matrix was damaged, creating vacancies, displacement cascades, and interstitial atoms [7,46,78]. These defects redistributed matrix atoms, including Cr atoms. The ability of Cr atoms to migrate and accumulate was determined by radiation-stimulated diffusion and defect concentration [58,60]. At a fluence of 1 × 10^16^ ion/cm^2^, matrix damage was insignificant, and defects had time to partially recombine. Cr atoms are weakly segregated toward defects. A fluence of 5 × 10^16^ ion/cm^2^ led to significant damage, segregation was more noticeable, and clustering could begin in defective zones. At the highest fluence of 1 × 10^17^ ion/cm^2^, severe matrix damage occurred, including the possible formation of amorphous regions. However, the line widths remained unchanged, indicating no signs of amorphization. In the defective zones, active segregation and growth of Cr clusters were occurring.

All of the above led to a decrease in the chromium content in the immediate vicinity of iron atoms, which resulted in an increase in the hyperfine magnetic field [31,79]. Furthermore, the iron content increased due to the ^57^Fe ions being implanted, thereby reducing the chromium content. This process was directly related to fluence magnitude. A similar dependence was previously explained by the clustering of Cr atoms [80], including that caused by the irradiation of Fe–Cr alloys with helium ions [31]. To some extent, the increase in the hyperfine field may also reflect the greater iron content due to the implanted ^57^Fe ions and the possible formation of chromium carbide compounds. In [38], the authors investigated the effect of the implantation of ^57^Fe ions with an energy of 1 MeV and a fluence of 5 × 10^16^ ion/cm^2^ on the properties of 12Cr18Ni10T stainless steel. The hyperfine magnetic field of the resulting martensite was 256 kOe, which was slightly higher than that for the CEMS spectrum of implanted CF8 steel at the same ion dose in the present study (254 ± 1 kOe). This was explained by the lower Cr content in the 12Cr18Ni10T steel matrix. In addition to the increase in the hyperfine magnetic field, the average angle between the incident γ-rays and the magnetization vector was determined in the CEMS spectra of CF8 steel samples at different fluences. It ranged from 58.7° to 63.5°, showing a very slight increase with increasing fluence. This result indicated that the fluence of the implanted Fe^57^ ions had an insignificant effect on the magnetic texture of the samples.

**Figure 4:**
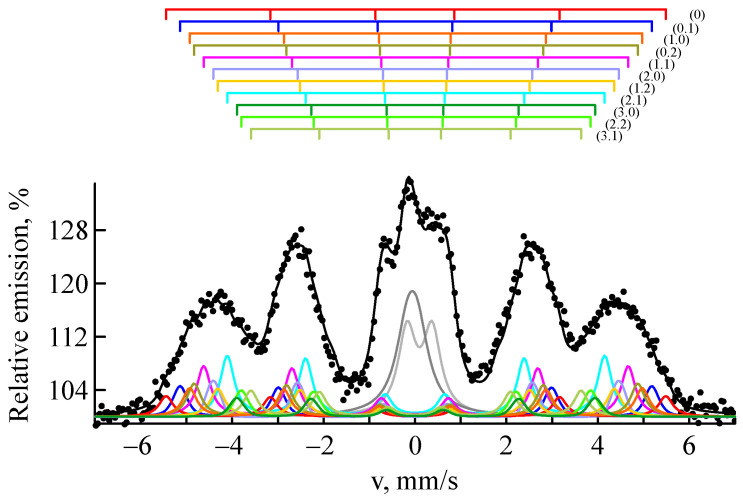
CEMS spectrum of CF8 steel sample after ^57^Fe ion implantation at a fluence of 1 × 10^17^ ion/cm^2^.

The distribution of the magnetic part p(H) in the CEMS spectra demonstrates probability peaks associated with Fe atomic configurations having different numbers of impurity atoms in the nearest sites of the bcc lattice. Figure 4 shows a fitting of the experimental CEMS spectrum of a steel sample implanted with ^57^Fe ions at a fluence of 1 × 10^17^ ion/cm^2^ using a set of several subspectra. Table 3 lists the Mössbauer parameters of the main subspectra describing α′-martensite. Thus, the sextet with the highest field (339 kOe) represents Fe atoms that are not surrounded by alloying element atoms (configuration 00). The two consecutive sextets (320 and 308 kOe) are associated with Fe atoms surrounded by one impurity atom in the two nearest coordination spheres, with the atom in the first coordination sphere reducing the field by 31 kOe (configuration 10), and in the second by 19 kOe (configuration 01). The influence of impurities located in the third and further coordination spheres is insignificant and is often neglected [71,77]. The isomer shift also varies with the number of nearest iron atoms. Atoms other than iron cause its decrease, indicating an increase in the s-like electron effective charge density at the Fe site [31,81]. Thus, analysis of the hyperfine magnetic field distribution reveals short-range ordering processes under irradiation.

**Figure 5:**
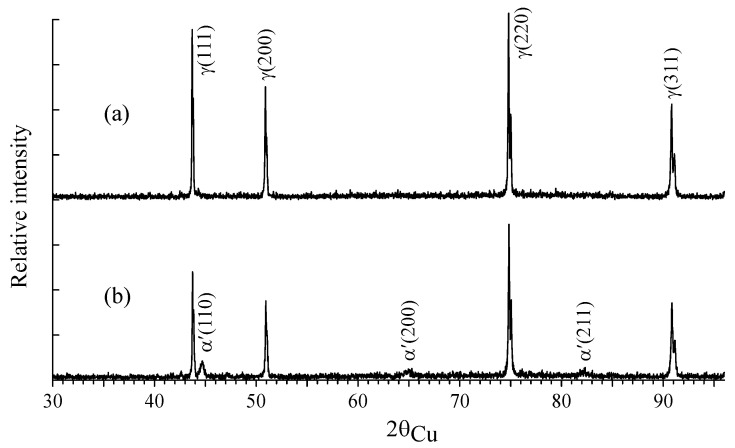
Diffraction patterns of CF8 samples before (**a**) and after ^57^Fe implantation to 1 × 10^17^ ion/cm^2^ (**b**).

Figure 5 shows the X-ray diffraction patterns of the samples before and after implantation at a fluence of 1 × 10^17^ ion/cm^2^. According to the X-ray diffraction data, the structure of the samples before implantation was γ-austenite with traces of carbon compounds (chromium carbides Cr_7_C_3_ and Cr_23_C_6_), undetectable by the methods of Mössbauer spectroscopy. The implantation of steel samples with ^57^Fe ions led to the appearance of α′-martensite reflections. Martensite was formed within the near-surface layer with a thickness limited by the projective range of ^57^Fe ions, and therefore α′-martensite was less pronounced at fluences of 1 × 10^16^ ion/cm^2^ and 5 × 10^16^ ion/cm^2^. In the sample implanted with ^57^Fe ions at a fluence of 1 × 10^17^ ion/cm^2^, its content reached ~10 wt.%. The bulk of the samples was dominated by γ-austenite, and no change in its lattice parameter (5.585 A) was detected as a result of implantation. Carbides Me_7_C_3_ and Me_23_C_6_ were also present in small amounts.

### 3.2. Thermal Treatment

**Figure 6:**
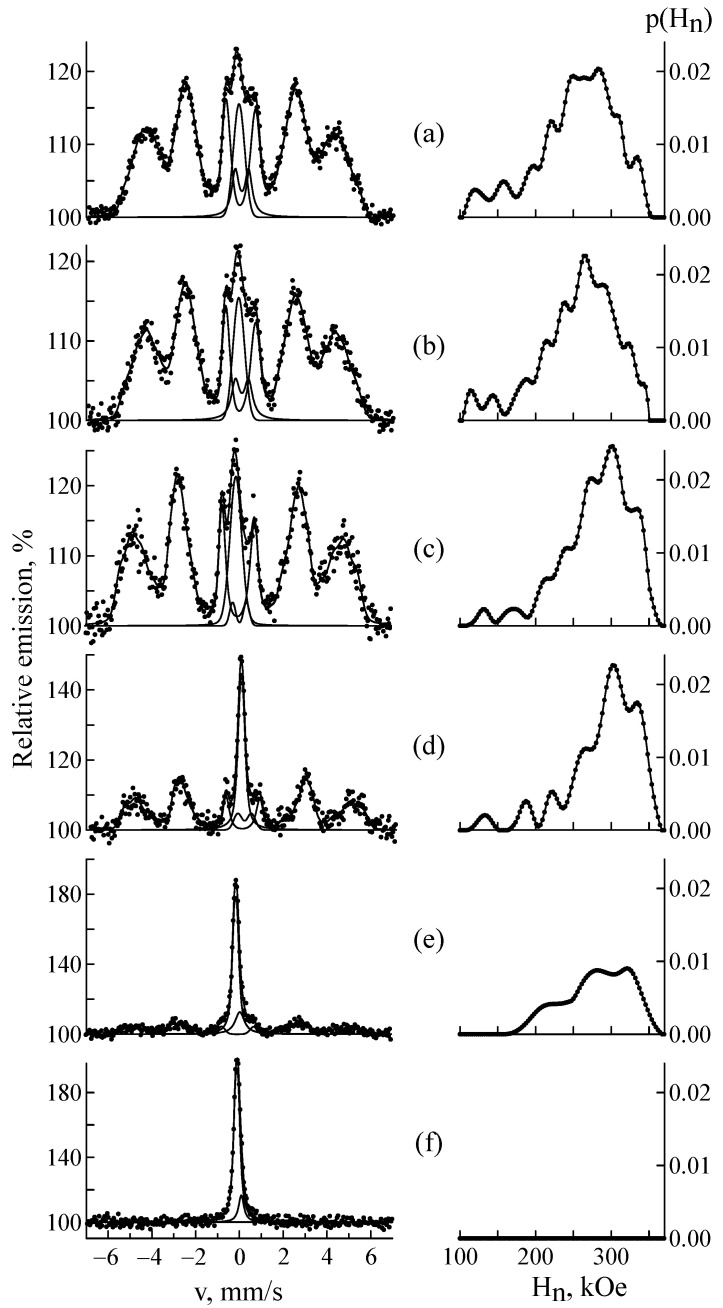
CEMS spectra and distributions of the hyperfine magnetic field p(H_n_) in CF8 steel implanted with ^57^Fe ions at a fluence of 5 × 10^16^ ion/cm^2^ before (**a**) and after annealing at a temperature of 300 (**b**), 600 (**c**), 700 (**d**), 800 (**e**) and 850 °C (**f**).

Figure 6 shows the CEMS spectra and distributions of the hyperfine magnetic field p(H_n_) for samples after implantation at a fluence of 5 × 10^16^ ion/cm^2^ followed by isochronal annealing at 300–850 °C. The results showed that the dynamics of the reverse α′ ⟶ γ-transformation was not observed at temperatures up to 600 °C. With increasing annealing temperature, the intensity of the ferromagnetic phase (α′-martensite) decreased while the relative intensity of the γ-austenite describing the doublet increased. The isomer shift in the doublet remained constant (~0.09 ± 0.01 mm/s), indicating that the electronic structure of austenite remained unchanged. The presumed carbide phase was also present; its parameters changed slightly, which was apparently due to variations in the proportions of its constituent carbides. Subsequently, at higher annealing temperatures, this phase will tend to dissolve back into austenite.

**Figure 7:**
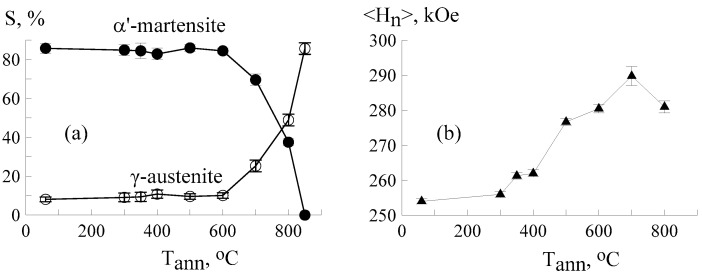
Relative intensity of α′-martensite, γ-austenite (**a**) and the average effective hyperfine field at ^57^Fe nuclei (**b**) in the CEMS spectra of CF8 steel as a function of the annealing temperature.

Figure 7a,b show the α′-martensite content and the average effective hyperfine magnetic field as a function of the annealing temperature. At temperatures up to 700 °C, an insignificant rate of the α′⟶γ-transformation was observed, and a noticeable increase in the average hyperfine magnetic field <H_n_> was noted, from 254 ± 1 to 279 ± 2 kOe. At the same time, the distribution of the magnetic field p(H_n_) (Figure 4) showed a gradual increase in the contribution from H_n_~335 kOe, which is explained by a decrease in the number of chromium atoms in the local environment of iron [32,79,82] and a more uniform bcc structure after annealing at these temperatures. At temperatures above 700 °C, an intense α′⟶γ-transformation occurred, induced by thermal motions of atoms [34], and <H_n_> decreased. After annealing at 850 °C, α′-martensite disappeared completely. The temperatures of the onset and end of the α′-martensite-to-γ-austenite transition determined for CF8 steel were somewhat higher than the transition temperatures of other steels of the Fe-Cr–Ni system [83,84]. This may be due to radiation-induced stabilization processes at high temperatures and pressures, caused by the accumulation of defects, internal stresses, and changes in the local chemical composition in the near-surface zone. In [85], the kinetics of the martensite-austenite transformation under thermal action (300–850 °C) were studied in CF8 duplex steel previously subjected to cold plastic deformation with a strain rate of ε = 55%. The dynamics of the reverse α′-martensite → γ-austenite transformation were not observed at temperatures up to 500 °C. An intense α′ ⟶ γ transition occurred in the temperature range of 600–700 °C. Practically no martensite was detected at a temperature of 800 °C.

## 4. Conclusions

The γ ⟶ α′-transformation during implantation of austenitic-ferritic steel CF8 with ^57^Fe ions at fluences of 1 × 10^16^, 5 × 10^16^ and 1 × 10^17^ ion/cm^2^, as well as the reverse α′ ⟶ γ–transformation under thermal treatment (300–850 °C) were studied using MS, CEMS, and XRD methods. It was found that implantation, which causes radiation damage with 36 dpa, results in the formation of α′-martensite. At higher fluences of implanted ions, the amount of α′-martensite increased, reaching 86 at% within the irradiated layer. The average hyperfine magnetic field <H> increased from ∼251 to ∼264 kOe with the growing fluence. It was also found that the presence of an impurity atom in the first coordination sphere of iron atoms reduced the field by 31 kOe and in the second by 19 kOe. A reverse transition of α′-martensite to γ-austenite was observed at annealing temperatures above 600 °C. After annealing at 850 °C, α′-martensite completely disappeared. The dependence of the average effective hyperfine magnetic field on annealing temperature was established.

## Figures and Tables

**Table 1:** Chemical composition of duplex stainless steel CF8, wt.%.

C	Si	Mn	P	S	Cr	Ni	Fe	
<0.08	<2	<1.5	<0.04	<0.04	18.0–21.0	8.0–11.0	balance	ASTM A743
0.07	1.6	1.0	-	-	19.4	8.6	69.3	Measured

**Table 2:** Phase composition of steel CF8 after ^57^Fe ion implantation determined from CEMS spectra.

Fluence, ion/cm^2^	S_α′_, %	S_γ_, %	S_MeC_, %
1 × 10^16^	58 ± 2	40 ± 2	2 ± 1
5 × 10^16^	86 ± 3	8 ± 1	6 ± 3
1 × 10^17^	85 ± 2	8 ± 2	7 ± 1

**Table 3:** Hyperfine parameters of α′-martensite subspectra of the CF8 steel sample after the implantation of ^57^Fe ions at a fluence of 1 × 10^17^ ion/cm^2^.

Atomic Configuration	Is, mm/s (±0.007)	H, kOe (±1)	S, % (±0.9)
0.0	0.062	339	4.9
0.1	0.053	320	4.3
1.0	0.030	308	3.5
0.2	0.020	302	9.1
1.1	0.015	288	10.9
2.0	0.018	276	6.2
1.2	0.000	268	6.7
2.1	−0.001	257	10.5
3.0	0.000	247	4.4
2.2	−0.011	238	6.4
3.1	−0.026	226	5.4

W (line width) was the same for all subspectra and equaled 0.37 ± 0.01 mm/s; the quadrupole splitting Qs was close to 0 mm/s.

## Data Availability

The original contributions presented in this study are included in the article. Further inquiries can be directed to the corresponding author.

## Supplementary Materials

The following supporting information can be downloaded at https://www.mdpi.com/article/10.3390/ma18215062/s1: Figure S1. Operation diagram of the first channel of the ^57^Fe implantation accelerator from the control software. (1—NEC MC-SNICS first channel ion source, 2—Source magnet, 3—Cascade generator, 4—Quadrupole lenses, 5—Analyzing magnet, 6—Rotary magnet, 7—Beam scanning and horizontal and vertical correction devices, 8—First channel implantation chamber).
