# Supplementary material for: Structural–Phase Transformations in Stainless Steel CF8 Under Ion Implantation and Thermal Treatment
Source: Materials (Basel). 2025 Nov 6;18(21):5062. doi: 10.3390/ma18215062 (PMC12608697; doi:10.3390/ma18215062)
Supplement: Supplementary file 1 [file materials-18-05062-s001.zip › materials-3903064-supplementary.pdf]

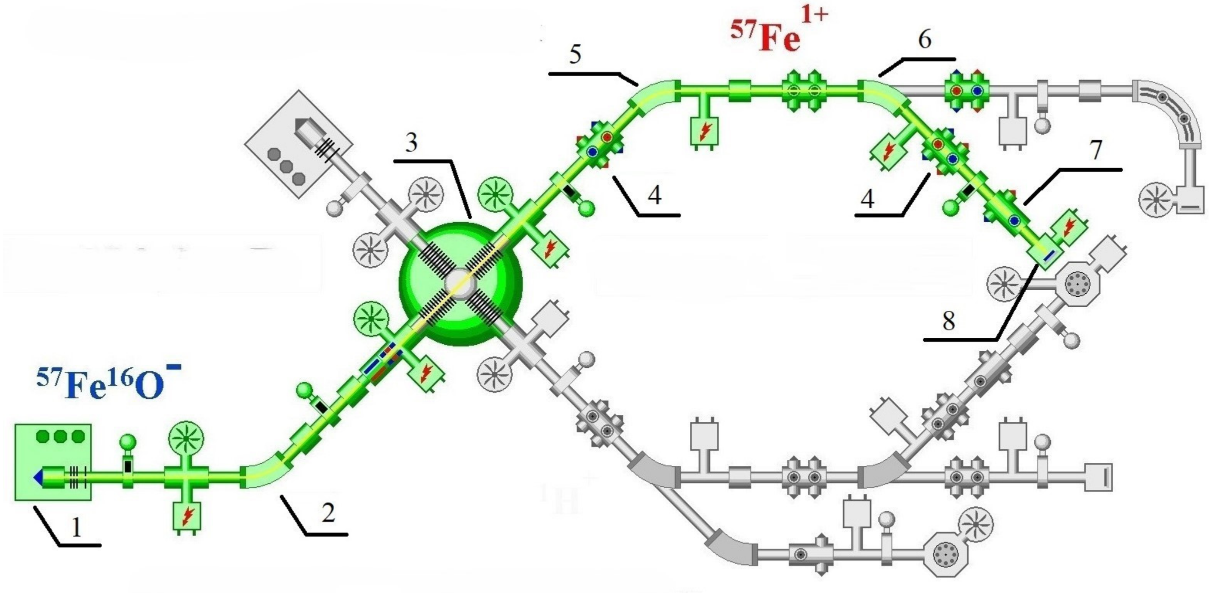

Figure S1. Operation diagram of the first channel of the ^{57}Fe implantation accelerator from the control software. (1—NEC MC-SNICS first channel ion source, 2—Source magnet, 3—Cascade generator, 4—Quadrupole lenses, 5—Analyzing magnet, 6—Rotary magnet, 7—Beam scanning and horizontal and vertical correction devices, 8—First channel implantation chamber).

A beam of ^{57}Fe oxide was extracted from the ion mass by a source magnet for further acceleration in a cascade generator. In the cascade generator's charge-exchange gas target, the molecular oxide compound was broken down into iron and oxygen. An analytical magnet selected the singly charged ^{57}Fe, and the beam was then transported by a bending magnet. Electrostatic quadrupole lenses were positioned along the transport channel, focusing the ion beam. The ion beam current was measured directly from the target. A metal ring was installed in front of the target, and a voltage of approximately -200 V was applied to it, blocking secondary electrons emitted from the target. This allowed for accurate measurement of the beam current at the target. An ORTEC 439 current integrator was used to measure the radiation dose, with data sent to the accelerator control PC for online dose conversion. The ion beam profile was formed as a normal distribution in the central part of the target. Beam sweeping ensured the ion beam size on the target (10 mm × 10 mm) reached the required dimensions, and beam positioning was adjusted using an electronic beam sweep and adjustment system directly in front of the channel implantation chamber. The spatial position of the beam during operation was monitored using a beam control system installed directly in front of the target.
